# Characterization data of chitosan-based films: Antimicrobial activity, thermal analysis, elementary composition, tensile strength and degree crystallinity

**DOI:** 10.1016/j.dib.2018.09.121

**Published:** 2018-10-03

**Authors:** Ana A. Escárcega-Galaz, Dalia I. Sánchez-Machado, Jaime López-Cervantes, Ana Sanches-Silva, Tomás J. Madera-Santana, Perfecto Paseiro-Losada

**Affiliations:** a Departamento de Biotecnología y Ciencia de los Alimentos, Instituto Tecnológico de Sonora, Ciudad Obregón, MX-85000 Sonora, México; b National Institute for Agricultural and Veterinary Research (INIAV), I.P., 655 Vairão, Vila do Conde, Portugal; c Centro de Investigación en Alimentación y Desarrollo, A.C. CTAOV, A.P. 1735, Hermosillo, MX-83304 Sonora, México; d Departmento de Química Analitica, Nutrición y Ciencia de Alimentos, Facultad de Farmacia, Universidad de Santiago de Compostela, 15782 La Coruña, Spain

**Keywords:** Chitosan, Thermal analysis, Honey-glycerol, Characterization data, Films, Degree crystallinity, Carbon-oxygen, *Klebsiella pneumoniae*, *Pseudomonas aeruginosa*, Differential scanning calorimetry

## Abstract

This set of raw and analyzed data are complement to the research article that is titled “Mechanical, structural and physical aspects of chitosan-based films as antimicrobial dressings” (Escárcega-Galaz et al., 2018) [1]. The mechanical, structural and biological properties of the chitosan-based films determine their potential application in biomedicine. The films were prepared from pure chitosan and in combination with honey or glycerol. Afterwards, the characterization data related to thermal analysis, elementary composition, tensile strength and degree crystallinity was collected. The data of the antimicrobial activity of the films correspond to *Klebsiella pneumoniae* and *Pseudomonas aeruginosa,* both isolated from cutaneous ulcers. This set of data indicate that the chitosan-based films possess biological and physicochemical characteristics for their application as antimicrobial dressings for their action when are used by direct contact during the treatment of cutaneous ulcers.

**Specifications table**

**Table:** 

Subject area	Carbohydrates, natural polyacids and lignins
More specific subject area	Polymers, biomedical,
Type of data	Tables, Figures, Text.
How data was acquired	Scanning electron microscope (SEM EVO LS15), texture analyzer (TA-XT2®), calorimeter (TA Instruments DSC Q100), X-ray Diffractometer (Phillips PW1710).
Data format	Analyzed
Experimental factors	Samples were only cut to an appropriate size to perform the tests.
Experimental features	The films of pure chitosan and others in mixture with honey-glycerol were characterized in relation to their antimicrobial activity, thermal analysis, elementary composition, tensile strength and degree crystallinity. All this to confirm its potential use as antimicrobial dressings during the healing of infected and chronic ulcers.
Data source location	Instituto Tecnológico de Sonora, Ciudad Obregón, Sonora, MX-85000, México.
	Centro de Investigación en Alimentos y Desarrollo, Hermosillo, Sonora, MX-83304, México.
	Universidad Santiago de Compostela, Santiago de Compostela 15782, La Coruña, Spain.
Data accessibility	The data are supplied with this article.
Related research article	Ana A. Escárcega-Galaz, Dalia I. Sánchez-Machado, Jaime López-Cervantes, Ana Sanches-Silva, Tomás J. Madera-Santana, Perfecto Paseiro-Losada (2018). Mechanical, structural and physical aspects of chitosan-based films as antimicrobial dressings. International Journal of Biological Macromolecules.
	doi:10.1016/j.ijbiomac.2018.04.149

**Value of the data**

• Data set for the characterization of six films based on chitosan and mixed with honey or glycerol.
• The characterization data of the chitosan films are related to the structural, mechanical and physical properties that favor its availability as an antimicrobial dressing.
• Between honey and chitosan there is a synergistic effect that improves their ability to eliminate infections and heal skin ulcers.
• This set of data of the essential properties of a biomaterial tends to strengthen the applications of chitosan as a film former for medical use, in addition to its biocompatibility and biodegradability.

## 1 Data

### 1.1 Antimicrobial activity *in vitro*

The composition of honey and its origin plays a very important role in its biological properties, specifically in the antimicrobial activity [2]. The honey is characterized for its sugars such as fructose, glucose, maltose and sucrose [3].

Table 1 shows the antimicrobial activity data of the chitosan-based films against *Klebsiella pneumoniae* and *Pseudomonas aeruginosa*, both are clinical origin. For the two microorganisms an area increment was observed. In the pure chitosan films, a similar behavior was observed between them and the addition of glycerol did not affect the increase in area values to a great extent. However, when honey was added the films increased their diameter considerably. Honey has high sugar content and low pH, these help prevent the growth of microorganisms [1].

**Table 1:** Area increase of chitosan films against *Klebsiella pneumonia* and *Pseudomonas aeruginosa*.

**Formulation**	***Klebsiella pneumoniae***		***Pseudomonas aeruginosa***	
	**Increase in area| (mm)**^a^	**% Increase in area**	**Increase in area (mm)**^a^	**% Increase in area**
Ch 1%	254.58 ± 2.08	9.13 ± 0.89	253.13 ± 1.46	8.51 ± 0.62
Ch 2%	255.98 ± 2.32	9.57 ± 0.99	252.14 ± 2.08	8.08 ± 0.89
Ch 3%	263.88 ± 1.55	13.08 ± 0.69	250.90 ± 1.23	7.55 ± 0.52
Ch 2% / Gly	255.03 ± 2.05	9.10 ± 0.87	251.93 ± 1.34	7.99 ± 0.57
Ch 2% / Honey	317.13 ± 8.02	36.73 ± 3.22	339.66 ± 11.22	45.60 ± 4.81
Ch 2% / Honey / Gly	336.68 ± 6.99	44.32 ± 2.99	338.24 ± 2.70	44.99 ± 1.16

a 233.27 mm^2^ initial area

### 1.2 Thermal analysis

The data of differential scanning calorimetry (DSC) measurements are shown in Table 2. The enthalpy variation shows with film formulation. By thermal analysis was possible found the temperature that resist the chitosan when is used pure or combined with other agents. These assays are indispensable for the process of manufacturing the biomaterial, and thus confirm that the chemical structure of chitosan remains intact, due it is not degraded during processing.

**Table 2:** Thermal events of chitosan films.

**Formulation**	**Tg (°C)**	**Cp (W/g)**	**Tm (°C)**	**ΔHm (J/g)**
Ch 1%	63.938	0.022764	169.384	77.03
Ch 2%	63.754	0.221945	170.563	121.9
Ch 3%	62.362	0.011058	134.968	107.6
Ch 2% / Gly	63.307	0.0175212	152.051	119.2
Ch 2% / Honey	62.755	0.0061252	144.266	164.8
Ch 2% / Honey / Gly	63.513	0.0077765	140.498	185.6

**Fig. 1:**
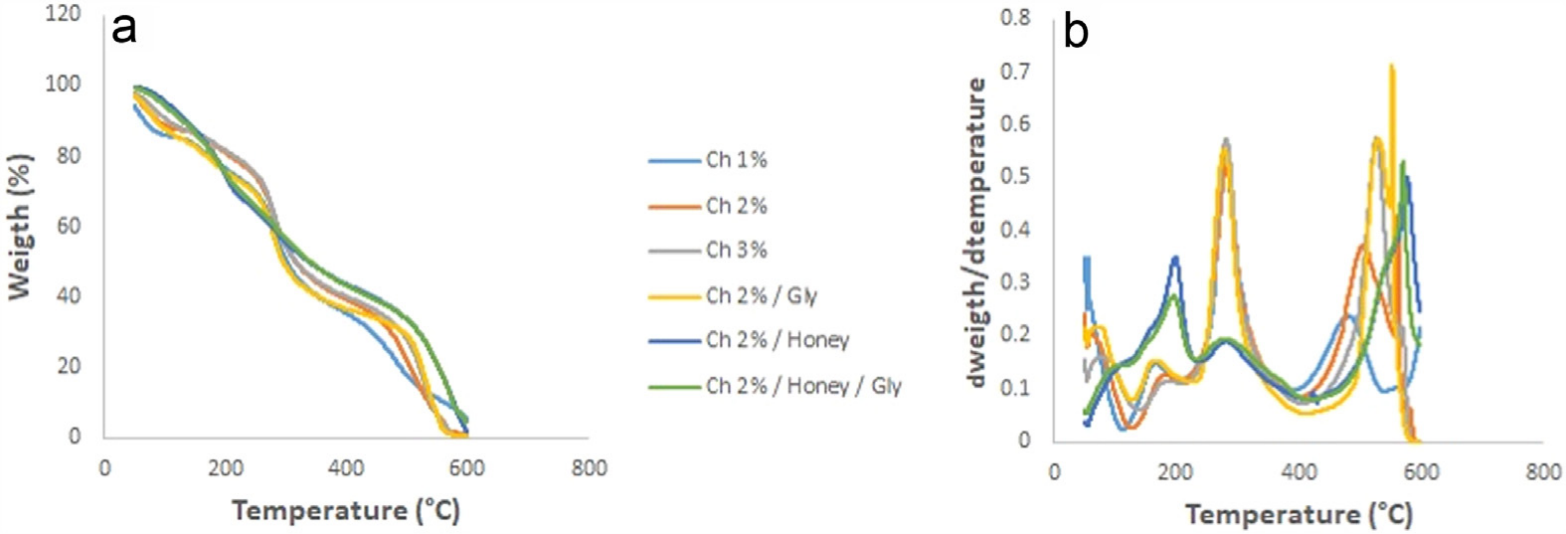
TGA thermograms (a) and derivative (b).

The thermogravimetric analysis (TGA) data revealed valuable information that confirms weight loss as the temperature increases. Fig. 1 shows the thermogram of the original data and the derivative.

### 1.3 Elementary composition

**Fig. 2:**
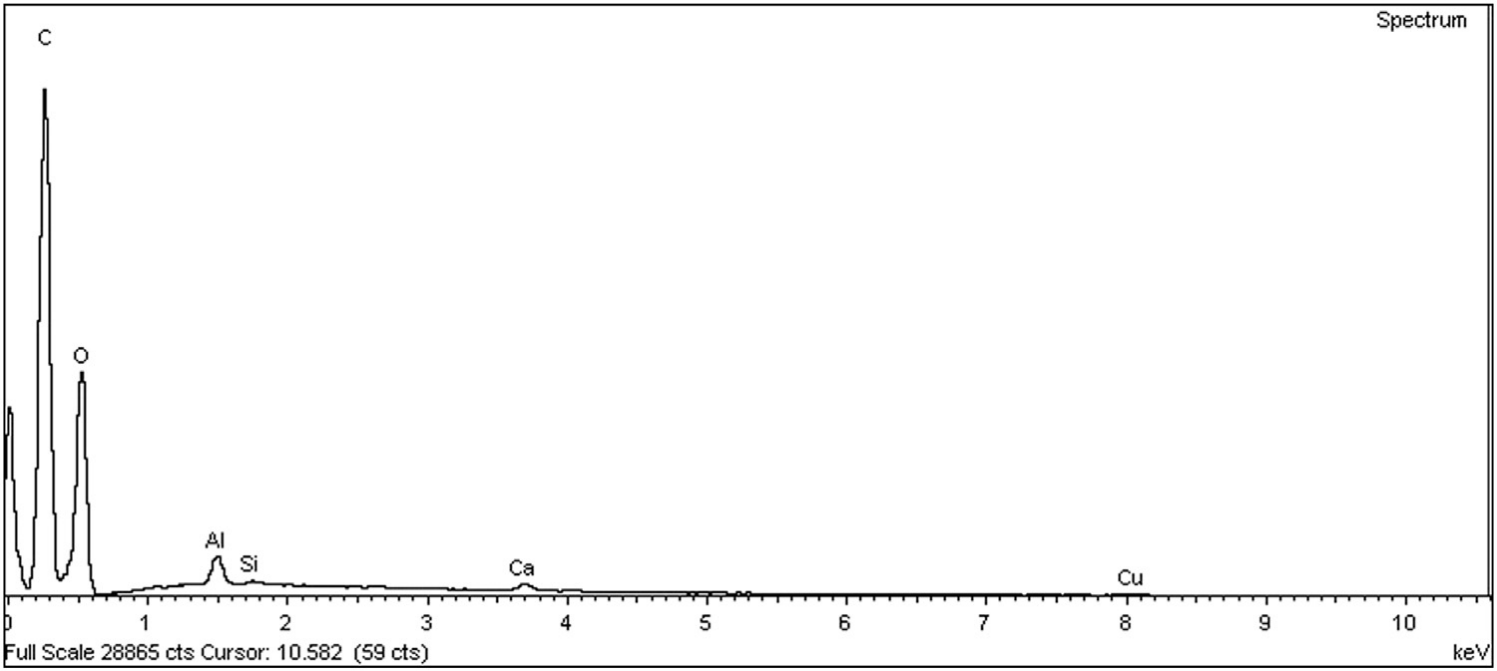
EDS spectrum of a 2% chitosan film.

Table 3 shows the carbon (C) and oxygen (O) content data of all the chitosan-based films formulations. Both elements are major components in all samples, around to 99%. Fig. 2 shows a characteristics energy-dispersive spectrum (EDS) of a 1% pure chitosan films.

**Table 3:** Carbon and oxygen data in chitosan-based films.

**Sample**	**Weight%**		**Atomic%**	
	**C**	**O**	**C**	**O**
Ch 1%	52.27 ± 0.18	47 ± 0.13	59.54 ± 0.17	40.19 ± 0.13
Ch 2%	53.31 ± 0.23	45.44 ± 0.62	60.66 ± 0.32	38.81 ± 0.49
Ch 3%	50.43 ± 0.05	48.64 ± 0.22	57.80 ± 0.04	41.86 ± 0.14
Ch 2% / Gly	51.75 ± 0.14	47.70 ± 0.18	58.98 ± 0.14	40.81 ± 0.17
Ch 2% / Honey	43.88 ± 4.47	54.52 ± 3.93	51.29 ± 4.47	47.94 ± 4.23
Ch 2% / Honey / Gly	38.31 ± 0.69	58.37 ± 0.76	45.94 ± 0.71	52.55 ± 0.71

In the films of pure chitosan and glycerol the behavior is similar, the variation was found in the films combined with honey where the carbon decreases and the oxygen increases, due to sugar being made up of carbon, hydrogen and oxygen.

The data in Table 4 show the trace elements found in the films such as Al, Ca, Si, Cu and Na. However, in all the formulations were found Al, Ca y Cu. Particularly in the films combined with honey, the aluminum content increased abundantly.

**Table 4:** Trace elements in chitosan-based films.

**Muestra**	**Weight %**					
	**Al**	**Si**	**Ca**	**Cu**	**K**	**Na**
Ch 1%	0.21 ± 0.04	0.11 ± 0.01	0.27 ± 0.01	0.19 ± 0.03	ND	ND
Ch 2%	0.53 ± 0.37	0.08 ± 0.01	0.3 ± 0.01	0.22 ± 0.08	ND	0.16 ± 0.01
Ch 3%	0.29 ± 0.15	ND	0.26 ± 0.01	0.26 ± 0.08	ND	ND
Ch 2% / Gly	0.05 ± 0.03	0.07 ± 0.01	0.24 ± 0.03	0.16 ± 0.02	ND	0.12 ± 0.01
Ch 2% / Honey	1.32 ± 0.29	ND	0.15 ± 0.01	0.25 ± 0.07	0.15 ± 0.01	ND
Ch 2% / Honey / Gly	2.12 ± 0.13	0.11 ± 0.01	0.31 ± 0.08	0.54 ± 0.26	0.20 ± 0.07	ND
	**Atomic %**					
**Muestra**	**Al**	**Si**	**Ca**	**Cu**	**K**	**Na**
Ch 1%	0.11 ± 0.02	0.06 ± 0.01	0.09 ± 0.01	0.04 ± 0.01	ND	ND
Ch 2%	0.27 ± 0.18	0.04 ± 0.01	0.10 ± 0.01	0.05 ± 0.01	ND	0.09 ± 0.01
Ch 3%	0.15 ± 0.07	ND	0.09 ± 0.01	0.05 ± 0.02	ND	ND
Ch 2% / Gly	0.03 ± 0.01	0.03 ± 0.01	0.08 ± 0.01	0.03 ± 0.01	ND	0.07 0.01
Ch 2% / Honey	0.69 ± 0.16	ND	0.05 ± 0.01	0.05 ± 0.02	0.05 ± 0.01	ND
Ch 2% / Honey / Gly	1.13 ± 0.07	0.06 ± 0.01	0.11 ± 0.02	0.12 ± 0.05	0.07 ± 0.02	ND

### 1.4 Tensile strength

**Fig. 3:**
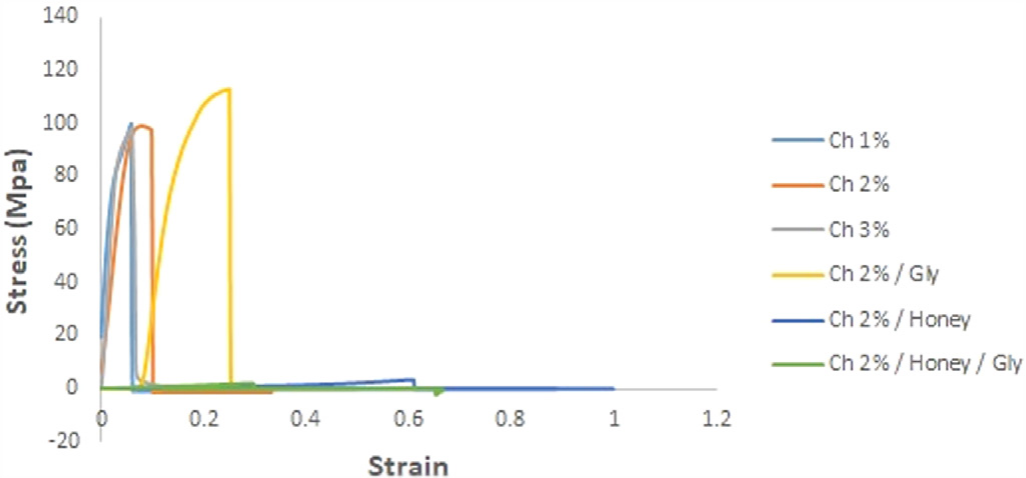
Tensile strength of the chitosan-based films.

With the data of the tensile strength the mechanical behavior of the chitosan-based films is complemented. All the formulations of the films showed a similar behavior. However, the force required to reach the rupture varied from one sample to another, see Fig. 3.

### 1.5 Degree of crystallinity

Table 5 shows the data of crystallinity of the pure chitosan-based films and their formulations, indicating that the grade of crystallinity decreases when the chitosan is combined with other agents. Consequently, all chitosan films were mainly in an amorphous state.

**Table 5:** Data of degree crystallinity of chitosan films.

**Sample**	**Crystal intensity**	**Intensity amorphous part**	**Total intensity**	**% Crystallinity**
Ch 1%	10567.25	119700.10	130267.40	8.1
Ch 2%	16054.56	158195.9	174250.4	9.2
Ch 3%	16933.84	168150.50	185084.30	9.1
Ch 2% / Gly	11323.5	129193.9	140517.3	8.0
Ch 2% / Honey	20228.23	337796.90	358025.1	5.6
Ch 2% / Honey / Gly	16521.16	315417.50	331938.60	4.9

## 2 Experimental design, materials, and methods

The chitosan-based films were prepared by the solvent evaporation technique. All the experiments were performed in triplicate minimum and the data analysis was carried out with the STATGRAPHICS Plus 5.1 software.

**Fig. 4:**
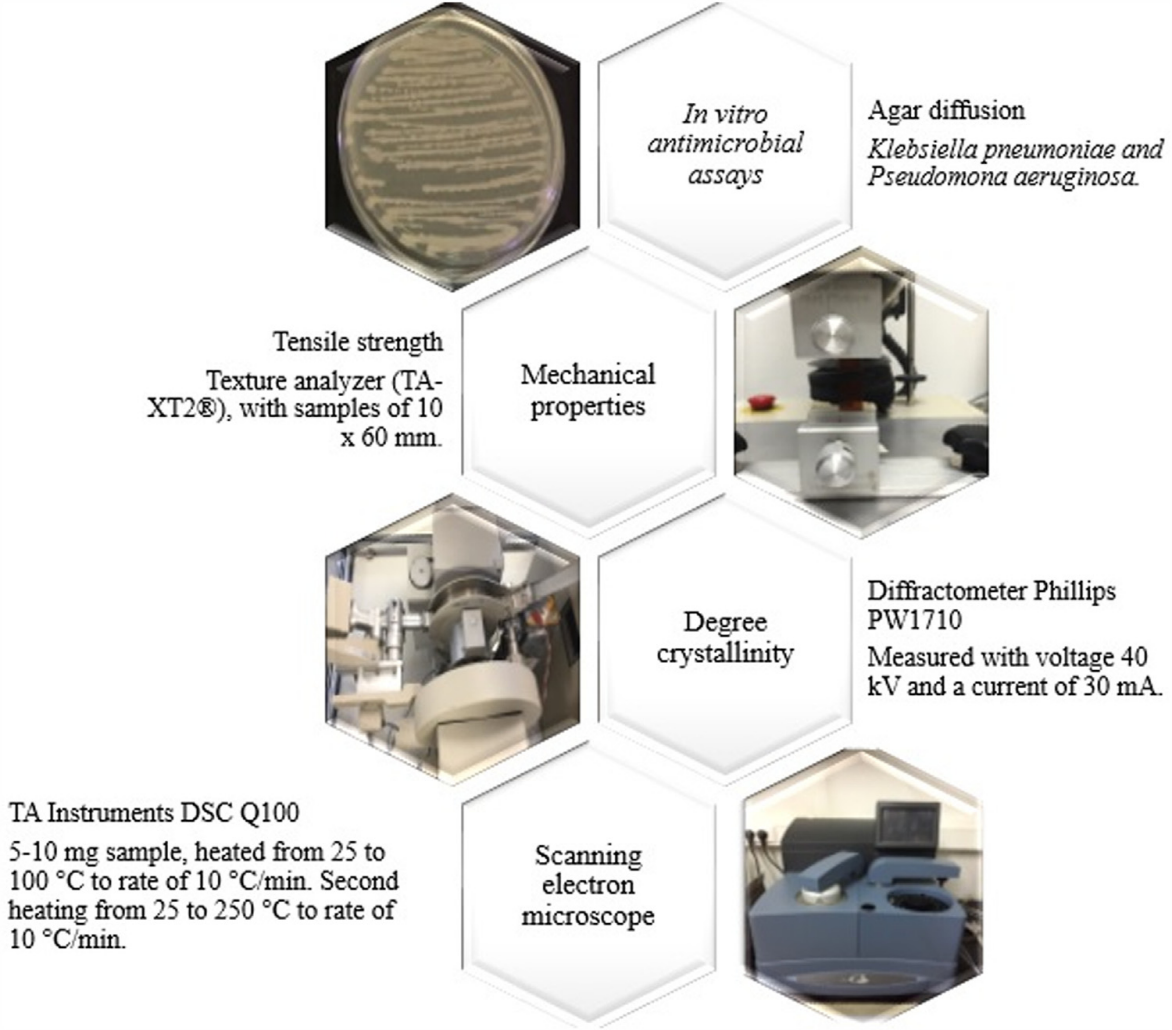
Methodology for the characterization of chitosan-based films.

Fig. 4 shows a flow chart of the methodologies for the characterization of chitosan-based films. Specifically, the details are described to evaluate the antimicrobial activity, thermal analysis by DSC, tensile strength and degree crystallinity.

Additionally, to evaluate the elementary composition was used a scanning electron microscope SEM EVO LS15 ZEISS brand (Oberkochen, Baden-Württemberg, Alemania) with an EDX detector. The sample was placed on a scanning support and the measurements were carried out with variable pressure vacuum (VP, 6.05 × 10-6 mbar), the voltage acceleration was 20 kV and the working distance (WD) was 8.5 mm.

Likewise, the thermogravimetric analysis (TGA) was carried out in TA Instruments Inc. Discovery (New Castle, DE, USA). The test was carried out with 20 mg of sample, which was heated from 50 to 600 °C at heating rate of 10 °C/min, under a nitrogen atmosphere with a flow of 60 ml/min.
